# Profiling of microRNAs in actinic keratosis and cutaneous squamous cell carcinoma patients

**DOI:** 10.1007/s00403-021-02221-2

**Published:** 2021-04-04

**Authors:** Aleksandra Dańczak-Pazdrowska, Jakub Pazdrowski, Adriana Polańska, Brittany Basta, Augusto Schneider, Michał J. Kowalczyk, Paweł Golusiński, Wojciech Golusiński, Zygmunt Adamski, Ryszard Żaba, Michal M. Masternak

**Affiliations:** 1 Department of Dermatology, Poznan University of Medical Sciences, Poznan, Poland; 2 Department of Head and Neck Surgery, Poznan University of Medical Sciences, Poznan, Poland; 3 Department of Head and Neck Surgery, The Greater Poland Cancer Centre, Poznan, Poland; 4 Department of Dermatology and Venereology, Poznan University of Medical Sciences, Poznan, Poland; 5 Department of Otolaryngolgy and Maxillofacial Surgery, University of Zielona Góra, Zielona Góra, Poland; 6 Department of Maxillofacial Surgey, Poznan University of Medical Sciences, Poznan, Poland; 7 College of Medicine, Burnett School of Biomedical Sciences, University of Central Florida, Orlando, FL USA; 8 Barbara Davis Center for Diabetes, , University of Colorado School of Medicine, Aurora, CO USA; 9 Faculdade de Nutrição, Universidade Federal de Pelotas, Pelotas, RS Brazil

**Keywords:** AK, CSCC, MiRNA, Cancer, Epigenetics

## Abstract

Actinic keratosis (AK) is a common skin lesion often defined as premalignant with more evidence indicating it as early stage of cutaneous squamous cell carcinoma (cSCC). The AK may remain stable, transform towards incisive cSCC or in some cases revert spontaneously. Several different underlying conditions can increase risk of cSCC, however, advanced age represents major risk of AK and its progression towards cSCC indicating increased risk during chronological aging. Importantly, AK and cSCC are characterized by similar genetic profile, which lead researchers to search for novel biomarkers allowing early detection. As skin sampling is often invasive and causes scaring, in the current study, we investigated a novel approach to establish potential blood circulating genetic markers in patients diagnosed with AK and cSCC. Based on clinical diagnosis and dermoscopy, we recruited 13 patients with AK (divided into two groups: the first included patients with no more than three lesions, the second group included patients with at least ten lesions) and two additional individuals diagnosed with cSCC. Deep sequencing analysis of serum circulating miRNAs detected a total of 68 expressed miRNAs. Further analysis indicated 2 regulated miRNAs for AK cohort and 12 miRNAs for cSCC patients, while there were 26 miRNAs differentially regulated between cSCC and AK patients. There was also one commonly regulated miRNA between AK and cSCC patients and ten miRNAs that were regulated in cSCC when compared with both control and AK patients. We did not observe any differences between the AK groups. In conclusion, our analysis detected in circulation some miRNA that were previously recognized as important in AK, cSCC, and other type of skin cancer supporting this approach as potential non-invasive diagnosis of AK and cSCC.

## Introduction

Actinic keratosis (AK) previously defined as a premalignant lesion, by many experts, is currently recognized as the early stage of cutaneous squamous cell carcinoma (cSCC). Both AK and cSCC share some similarities in genetic profile and histopathological features [1–4]. Importantly, NMSC represents one of the most common types of cancer, especially among Caucasians, with 1 million new cases per year in the United States, and cSSC also represents the second most common skin malignancy (20% of all nonmelanoma skin cancers, NMSCs), although most of statistical data seem to be underestimated [5, 6]. All NMSCs are associated with a substantial morbidity but in contrast to basal cell carcinoma, cSCC is characterized by much higher risk of metastases [5, 6]. The vast majority of cSSC arise from AK, and the prevalence of AK was estimated at 11–25% with significant increase during chronological age [1, 2, 4]. All those observations indicate AK as one of the most common dermatological condition and a very important health care concern especially for growing elderly populations.

By analogy with histopathological classification of other intraepithelial neoplasia, AK is classified into three stages: keratinocytic intraepidermal neoplasma (KIN) I–III, in which KIN I represents the mildest stage of AK with atypical keratinocytes located only in the lower third of epidermis, while KIN III describes involvement of full epidermis thickness. Importantly, AK occurs mainly on a sun damaged skin of head and neck called field cancerization. In this particular area, disseminated atypical basal keratinocytes can be observed despite the lack of clinically obvious AK [1, 2, 4]. Given that cSCC can appear within clinically silent area or KIN I, so called “differentiated pathway” of transformation which is believed to be more common and more aggressive than progression from KIN III, and on the other hand some AK lesions may undergo spontaneous clinical regression, currently it cannot be anticipated which AK would eventually transform into cSCC based on clinicopathological findings [2, 7]. Recent studies suggest that the genes *EIF4EBP1, SNX17, PRPF4, NXT1, and UBA5* can contribute to progression of AK into cSCC [8]. However, it is argued that the estimated annual risk of progression from AK to cSCC for an individual lesion is rather low, with assessed risk around 16%, yet the cumulative risk for patients with multiple lesions is much more significant reaching up to 80%, and the relative risk of transformation increases for those with more than five lesions [1–4]. Therefore, collectively data indicate that the multiple AK lesions might constitute one of the most important risk factor of progression to cSCC.

MicroRNAs (miRNAs) have an emerging role in the understanding of cancer pathophysiology and diagnosis. miRNAs comprise short (~ 22 nt) single strand RNA molecules which are able to promote target mRNA cleavage and translation repression [9]. Understanding the regulation of oncogenes and tumor-suppressor genes is pivotal for diagnosis and treatment of cancer. In this context, it has been demonstrated that miRNAs can regulate cancer related genes [10]. miRNA profiling has proven effective in distinguishing several types of cancer, including breast cancer, glioblastoma, colorectal cancer, lung, and hepatocellular carcinoma [10]. Therefore, some miRNAs have been classified as oncogenic and tumor suppressor as well. One study identified that at least three different miRNAs were commonly regulated in breast, colon, lung, pancreas, prostate, and stomach cancers [11]. This evidence suggests miRNAs as involved in the development and progression of cancer. Our group has also shown before a miRNA signature in head and neck and colorectal cancer samples [12–14]. In cSCC, some miRNAs differentially regulated include miR-21, miR-205, miR-365, miR-34a, and miR-125b [15]. Additionally, miR-193b and miR-199b have a gradual regulation throughout the malignant evolution of AK to cSCC, while miR-19 and miR-126 seems early stage specific markers of AK [16]. Therefore, better understanding of specific miRNAs regulated during the evolution of AK to cSCC is required and will also provide confirmation of the role of miRNAs in this context.

However, miRNAs do not have an exclusively intracellular role and are found outside the cell, including in serum [17–19]. miRNAs can reach the extracellular environment through passive leakage from damaged cells or active secretion in exosomes [20]. In this sense, miRNAs in serum can target oncogenes/tumor-suppressor genes in distant or adjacent target cells [20]. Additionally, the pattern of serum miRNAs can be used as tool for non-invasive diagnosis [17, 21, 22]. Although some papers describing serum miRNAs in melanoma patients can be found [23], no literature for serum miRNAs in AK and cSCC can be found. In this context, the goal of our research was to assess if there is any difference in the expression of circulating miRNA within two groups of patients with AK: first one presented with no more than three lesions of AK, and the second with at least ten lesions.

## Materials and methods

### Patient selection

Overall, 13 patients with AK within the skin of the head were recruited into this observational study based on clinical diagnosis and dermoscopy. The assessments were performed independently by two experienced dermatologist: ADP and AP. Including criteria for AK patients were: (a) Patient with 1–3 lesions or patients with at least 10 lesions; and (b) Otherwise healthy. Excluding criteria for AK patients were: (a) Patient with comorbidities; (b) Patients with more than 3 and less than 10 lesions; (c) Patients with clinical features that can suggest transformation to cSCC (induration, prominent inflammation, size > 1 cm in diameter, rapid growth, bleeding, and ulceration); d) Patients with previous NMSC. Patients were divided into two groups: the first group included patients with no more than three lesions of AK (6 patients, 4 men and 2women, mean age 73.2 years), the second group included patients with at least ten lesions of AK (7 patients, 6 men and 1 woman, mean age 84.3 years). The diagnosis of AK has not been confirmed by histopathological examination, but it is consistent with the recommendations of the International League of Dermatological Societies [24].

Additionally, two male patients with cSCC within the skin of head, otherwise health, were recruited to the study (mean age 73.5 years) and four healthy subjects (3 men and 1 woman, mean age 72,2 year). The diagnosis of cSCC was based on histopathological examination. Excluding criteria for cSCC patients: (i) Patient with comorbidities; (ii) Patients with the history or diagnosis during the recruitment with other cutaneous/noncutaneous neoplasm. Excluding criteria for healthy subjects: (a) comorbidities; (b) diagnosis of past or present oncological diseases. All patients included in the study were Caucasians with skin type II according to the Fitzpatrick classification. From all selected participant 5 ml of whole blood sample was collected. Following centrifugation, serum samples were stored at – 80 °C.

The study was approved by a local bioethical committee (Poznan University of Medical Sciences, no 589/19). All patients gave written consent.

### RNA extraction and miRNA library preparation

The samples were removed from the -80° C freezer and homogenized with Qiazol (Qiagen, Valencia, CA, USA) using 0.5 mm zirconium oxide beads in the Bullet Blender 24 (Next Advance, Averill Park, NY, USA). Total RNA was extracted using a commercial column purification system (miRNeasy Mini Kit, Qiagen) and on-column DNase treatment (RNase-free DNase Set, Qiagen) following manufacturer's instructions.

MicroRNA libraries were prepared using the NEBNEXT Small RNA Library Prep Set for Illumina (New England Biolabs) following the manufacturer's instructions and adjusted by Matkovich, et al. [25]. Briefly, small RNAs from 1 μg of total RNA were ligated with 3′ and 5′ adapters, followed by reverse transcription to produce single-stranded cDNAs. Samples were then amplified by PCR in 14 cycles using indexes to allow all individual libraries to be processed in a single flow cell lane during sequencing. The amplified libraries were size-selected and purified in a 6% agarose gel.

The quantity and quality of miRNA libraries were determined using BioAnalyzer and RNA Nano Lab Chip Kit (Agilent Technologies, Santa Clara, CA, USA), and the samples were combined in a single microtube and submitted to sequencing on a HiSeq 2500 instrument (Illumina Inc.).

### miRNAs libraries analysis and statistical analyses

Alignment and quantification of miRNA libraries was performed using sRNAtoolbox as described before [26]. Statistical analyses of differentially expressed miRNAs were performed using EdgeR [27] on the R software (3.2.2) and miRNAs with a FDR < 0.05 and FC > 2.0 were considered as up-regulated; and FDR < 0.05 and FC < 0.50 were considered as down-regulated.

### miRNAs target prediction and enriched pathways and GO terms

The mirPath tool (version 3.0) was used to predict target genes of the differentially regulated miRNAs using the microT-CDS v. 5.0 database [28] and for retrieving KEGG molecular pathways [29, 30], considering *P* values lower than 0.05 as significant for pathway enrichment.

## Results

**Fig. 1:**
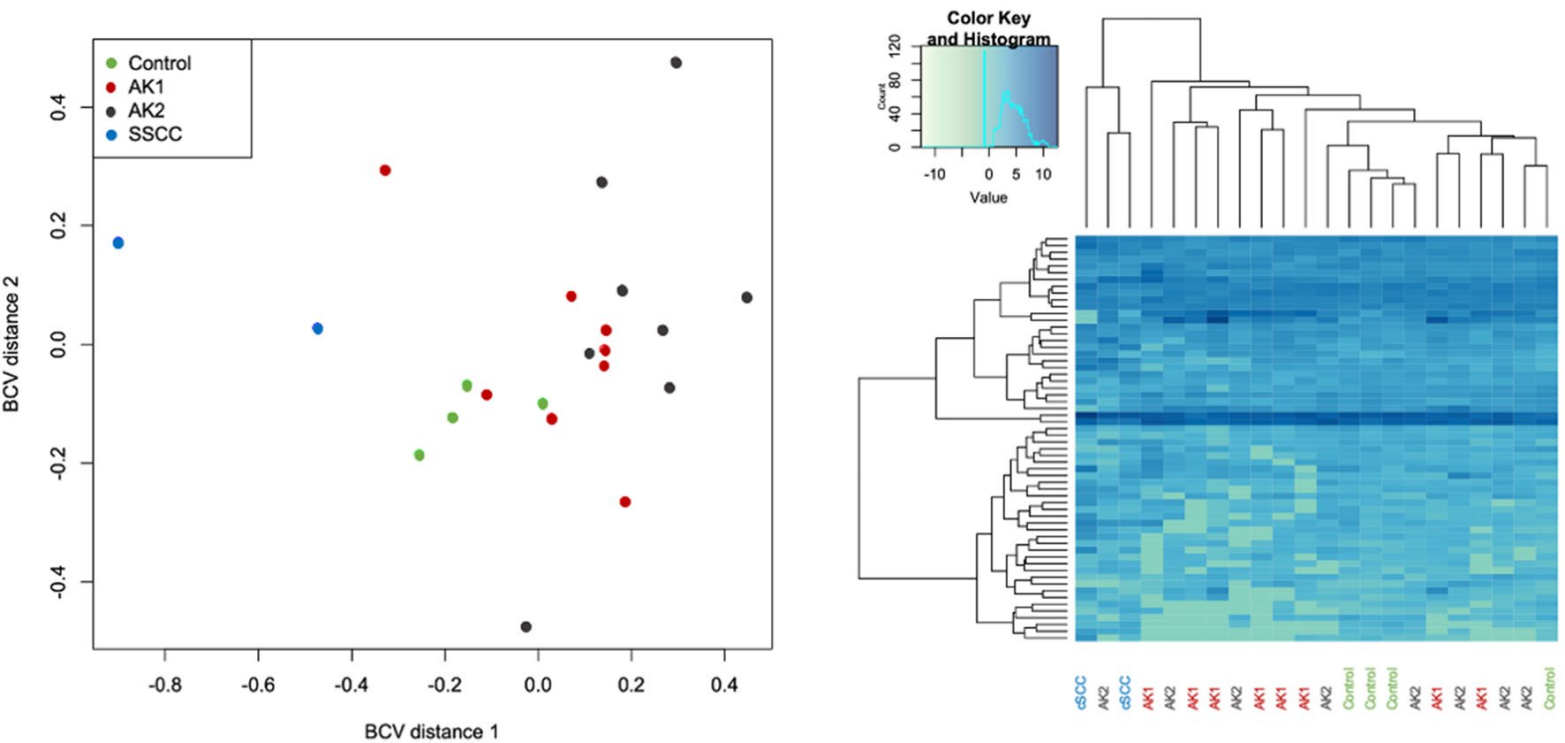
**a** Principal component analysis and **b** unsupervised hierarchical clustering of the 60 miRNAs in skin samples from patients diagnosed with cancer

After sequencing and processing of the RNASeq data, from an average of 9,650,146.05 ± 814,659.28 raw reads, 5,503,515.68 ± 337,285.95 adapter cleaned reads/sample were obtained with 89% alignment rate to the human genome (hg19). A total of 68 different known miRNAs were identified in the serum of patients. PCA analysis and unsupervised hierarchical clustering of the samples indicate some distinction among groups (Fig. 1).

**Fig. 2:**
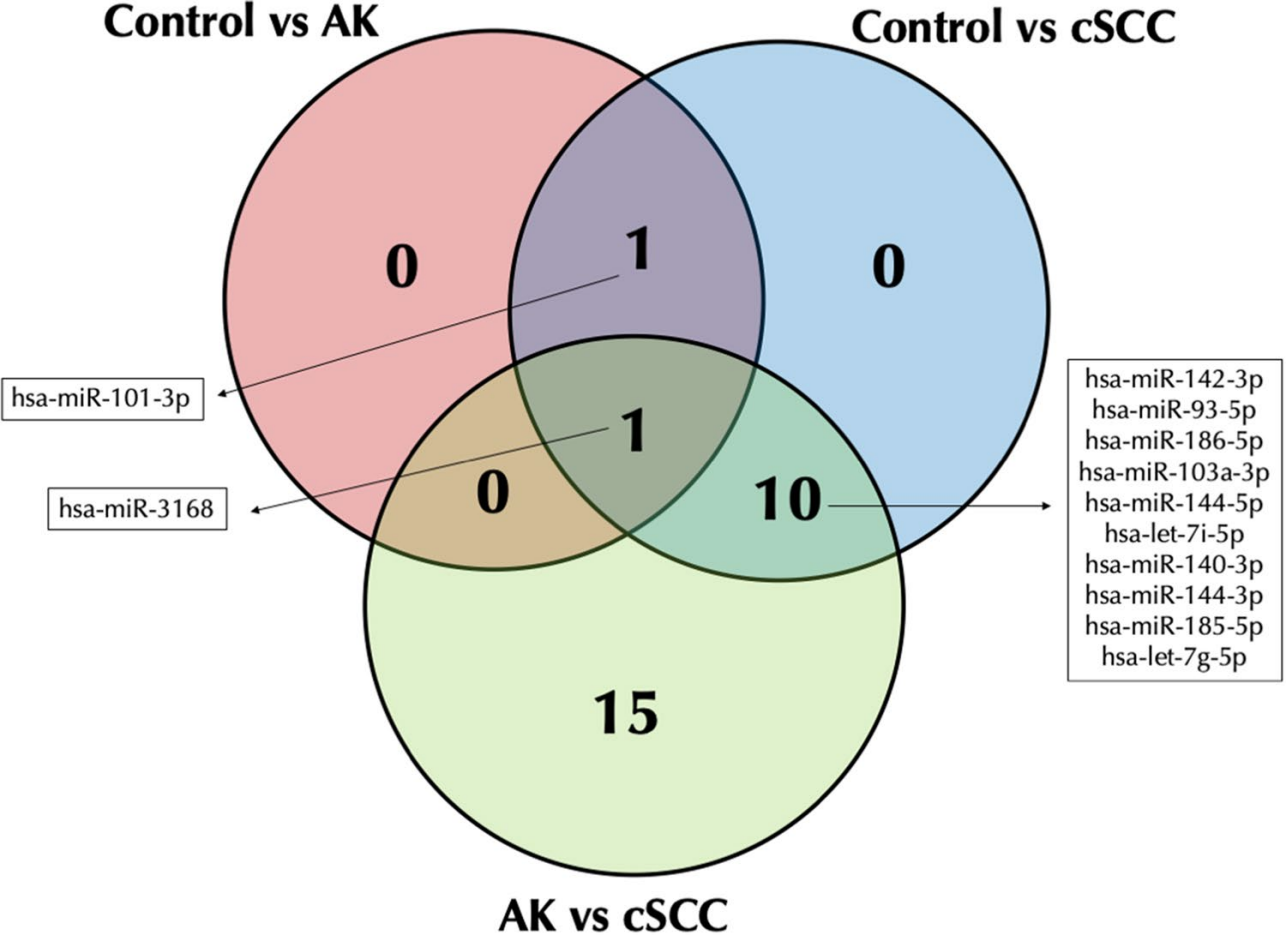
Venn diagram of miRNA expression among groups

Based on the significant overlap among AK groups and the absence of differentially expressed miRNAs, we combined both AK groups in a single AK group. When comparing expression profile of AK patients, we observed 2 regulated miRNAs (Table 1), 12 miRNAs for cSCC patients (Table 1), and 26 miRNAs between cSCC and AK patients (Table 1). There was 1 commonly regulated miRNA between AK and cSCC patients and 10 miRNAs that were regulated in cSCC compared to both control and AK patients (Fig. 2).

**Table 1:** -MicroRNAs differentially expressed in the serum of AK, SSCC, and healthy control patients

miRNA^a^	Control	AK	FC^b^	*P* value	FDR^c^
hsa-miR-3168	56.97 ± 13.64	328.18 ± 67.67	5.74	0.0003	0.020
hsa-miR-101-3p	8.55 ± 1.19	24.18 ± 2.27	2.75	0.0013	0.046

	Control	SSCC	FC	*P* value	FDR
hsa-miR-144-3p	6.13 ± 2.32	98.46 ± 79.96	15.53	0.0002	0.021
hsa-miR-140-3p	33.25 ± 13.48	246.74 ± 97.35	7.33	0.0004	0.025
hsa-let-7 g-5p	66.01 ± 14.93	427.39 ± 337.91	6.46	0.0012	0.025
hsa-let-7i-5p	170.85 ± 26.99	943.27 ± 653.10	5.52	0.0008	0.025
hsa-miR-103a-3p	19.70 ± 6.89	154.39 ± 78.40	7.74	0.0007	0.025
hsa-miR-185-5p	54.67 ± 25.15	428.64 ± 353.85	7.80	0.0013	0.025
hsa-miR-93-5p	10.08 ± 1.86	83.64 ± 70.04	8.35	0.0014	0.025
hsa-miR-101-3p	28.20 ± 3.93	190.05 ± 172.78	6.75	0.0030	0.035
hsa-miR-142-3p	0.00 ± 0.00	10.53 ± 7.96	337.17	0.0029	0.035
hsa-miR-144-5p	0.71 ± 0.58	19.87 ± 16.08	22.52	0.0024	0.035
hsa-miR-186-5p	9.74 ± 3.77	89.14 ± 75.54	8.92	0.0030	0.035
hsa-miR-3168	184.41 ± 44.06	16.09 ± 15.53	0.08	0.0044	0.047

	AK	SSCC	FC	*P* value	FDR
hsa-miR-93-5p	0.75 ± 0.29	26.82 ± 23.40	31.66	2.15E–07	2.64E–05
hsa-miR-185-5p	18.04 ± 2.40	137.38 ± 117.11	7.63	2.83E–06	1.74E–04
hsa-miR-3168	407.19 ± 82.16	4.58 ± 4.58	0.01	4.63E–06	1.90E–04
hsa-miR-451a	1065.99 ± 103.22	4445.68 ± 2785.16	4.17	9.03E–06	2.78E–04
hsa-miR-103a-3p	5.1 ± 1.18	48.23 ± 27.58	9.09	2.87E–05	0.001
hsa-miR-652-3p	0.57 ± 0.24	11.94 ± 9.86	17.50	3.86E–05	0.001
hsa-miR-107	1.55 ± 0.35	13.00 ± 7.18	7.96	0.0001	0.001
hsa-let-7i-5p	71.41 ± 10.14	299.23 ± 219.59	4.17	0.0001	0.001
hsa-miR-144-3p	2.81 ± 0.68	31.50 ± 26.74	11.15	0.0001	0.001
hsa-miR-17-5p	0.17 ± 0.16	10.93 ± 10.52	54.10	0.0001	0.001
hsa-let-7 g-5p	24.03 ± 7.39	136.60 ± 112.29	5.63	0.0006	0.006
hsa-miR-140-3p	11.91 ± 3.48	76.42 ± 35.50	6.35	0.0009	0.008
hsa-miR-144-5p	0.51 ± 0.21	6.34 ± 5.60	10.18	0.0009	0.008
hsa-miR-186-5p	3.41 ± 1.27	28.61 ± 25.19	8.18	0.0010	0.008
hsa-miR-142-3p	0.12 ± 0.11	3.32 ± 2.89	19.50	0.0018	0.013
hsa-miR-425-3p	0.14 ± 0.12	2.63 ± 1.49	15.21	0.0019	0.013
hsa-miR-22-3p	41.30 ± 3.23	107.65 ± 32.61	2.61	0.0025	0.015
hsa-miR-183-5p	0.52 ± 0.26	6.50 ± 6.09	10.71	0.0028	0.016
hsa-miR-363-3p	6.27 ± 1.47	33.85 ± 25.38	5.31	0.0031	0.017
hsa-miR-16-5p	5.80 ± 1.19	33.95 ± 29.19	5.72	0.0034	0.018
hsa-miR-629-5p	2.05 ± 0.75	15.36 ± 8.53	7.08	0.0037	0.018
hsa-miR-23a-3p	1.64 ± 1.00	17.76 ± 3.39	10.34	0.0050	0.022
hsa-miR-26b-5p	6.66 ± 1.50	30.60 ± 21.11	4.42	0.0059	0.025
hsa-miR-106b-3p	6.46 ± 1.44	31.58 ± 26.15	4.82	0.0079	0.032
hsa-miR-122-5p	509.71 ± 211.92	14.04 ± 14.04	0.03	0.0081	0.032
hsa-miR-15b-5p	1.26 ± 0.42	8.18 ± 4.71	6.02	0.0094	0.036

^a^miRNAs are expressed as reads per million (rpm). miRNAs with less than 3 rpm in more than 50% of the samples were removed from analysis

^b^Fold change in tumor compared to healthy tissue

^c^False discovery rate. Only miRNAs with FDR lower than 0.05 were considered as significantly regulated

**Fig. 3:**
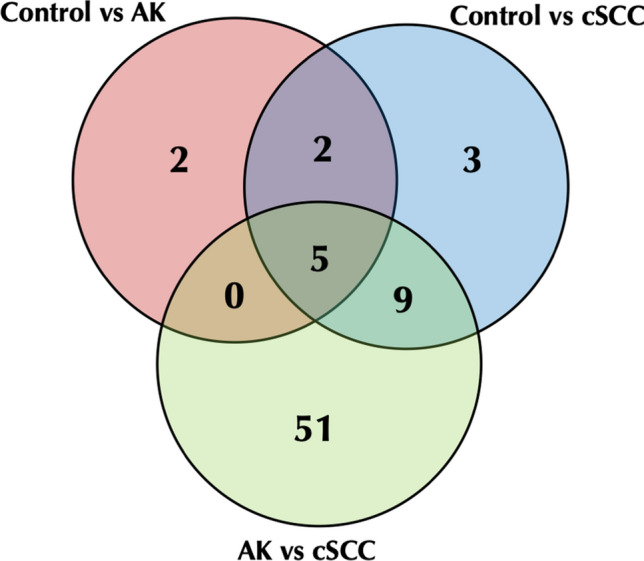
Venn diagram of regulated KEGG pathways among groups

Among the regulated pathways by target genes of these miRNAs, we observed that TGF-beta signaling pathway, cAMP signaling pathway, and signaling pathways regulating pluripotency of stem cells were commonly regulated in AK and cSCC samples (Table 2 and Fig. 3). Among the top regulated pathways, we also observed signaling pathways regulating pluripotency of stem cells, pathways in cancer and proteoglycans in cancer, further confirming the role of the miRNAs in the regulation of cancer pathophysiology (Tables 3 and 4).

**Table 2:** Pathways of target genes from miRNAs differentially expressed between control and AK tissue

KEGG pathway	*P* value	Genes	miRNAs
Drug metabolism—cytochrome P450	1.11E–16	1	1
Morphine addiction	0.002	11	2
Signaling pathways regulating pluripotency of stem cells	0.003	6	1
TGF-beta signaling pathway	0.004	9	1
Endocrine and other factor-regulated calcium reabsorption	0.013	1	1
Mucin type O-glycan biosynthesis	0.019	1	1
Axon guidance	0.047	15	1
Dopaminergic synapse	0.049	17	1
cAMP signaling pathway	0.050	24	1

**Table 3:** Pathways of target genes from miRNAs differentially expressed between control and SSCC tissue

KEGG pathway	*P* value	Genes	miRNAs
Signaling pathways regulating pluripotency of stem cells	1.14E–09	55	5
Proteoglycans in cancer	2.39E–06	72	4
Axon guidance	1.89E–04	45	4
Gap junction	0.002	23	4
Mucin type O-glycan biosynthesis	6.29E–07	6	4
cAMP signaling pathway	0.049	58	3
Dopaminergic synapse	0.008	46	3
Adrenergic signaling in cardiomyocytes	0.033	42	3
Glutamatergic synapse	0.022	33	2
ErbB signaling pathway	0.028	29	2
Amphetamine addiction	3.30E–04	27	2
TGF-beta signaling pathway	0.010	24	2
Transcriptional misregulation in cancer	0.047	20	2
ECM-receptor interaction	1.11E–16	8	2
Glycosaminoglycan biosynthesis—chondroitin/dermatan sulfate	0.013	4	2
Glioma	0.028	17	1
Fatty acid metabolism	4.86E–05	3	1
Fatty acid biosynthesis	1.78E–15	1	1
Drug metabolism—cytochrome P450	3.33E–08	1	1

**Table 4:** Pathways of target genes from miRNAs differentially expressed between AK and SSCC tissue

KEGG pathway	*P* value	Genes	miRNAs
Pathways in cancer	2.06E−05	188	24
PI3K–Akt signaling pathway	0.001	155	24
Regulation of actin cytoskeleton	0.001	103	23
Focal adhesion	0.003	99	23
Signaling pathways regulating pluripotency of stem cells	1.04E−05	75	23
FoxO signaling pathway	2.84E−04	71	23
AMPK signaling pathway	3.57E−04	68	23
Dopaminergic synapse	0.008	67	23
Tight junction	0.004	65	23
ErbB signaling pathway	3.57E−04	51	23
MAPK signaling pathway	0.003	119	22
Proteoglycans in cancer	3.95E−12	117	22
Ras signaling pathway	0.004	101	22
Rap1 signaling pathway	0.011	95	22
cAMP signaling pathway	0.007	94	22
cGMP–PKG signaling pathway	0.005	80	22
Adrenergic signaling in cardiomyocytes	1.12E−05	74	22
Insulin signaling pathway	0.038	65	22
Neurotrophin signaling pathway	0.003	63	22
Platelet activation	0.005	63	22
Small cell lung cancer	0.011	44	22
Estrogen signaling pathway	0.044	43	22
mTOR signaling pathway	3.49E−05	40	22
Viral carcinogenesis	0.003	74	21
Axon guidance	5.18E−06	69	21
Thyroid hormone signaling pathway	5.18E−06	66	21
Glutamatergic synapse	6.88E−05	61	21
Choline metabolism in cancer	0.023	51	21
T-cell receptor signaling pathway	0.038	51	21
Prostate cancer	0.004	47	21
Melanoma	0.003	39	21
Renal cell carcinoma	0.006	38	21
Prolactin signaling pathway	0.002	37	21
Glioma	1.10E−04	36	21
Pancreatic cancer	0.002	36	21
Non-small cell lung cancer	0.008	30	21
Oxytocin signaling pathway	1.05E−04	84	20
Hippo signaling pathway	1.51E−05	76	20
Wnt signaling pathway	3.87E−04	72	20
Sphingolipid signaling pathway	0.001	62	20
Long-term potentiation	3.57E−04	41	20
Chronic myeloid leukemia	0.013	37	20
Bacterial invasion of epithelial cells	0.038	36	20
Cholinergic synapse	0.030	54	19
Amoebiasis	0.046	46	19
TGF-beta signaling pathway	3.49E−05	45	19
Insulin secretion	0.044	41	19
Phosphatidylinositol signaling system	0.005	40	19
p53 signaling pathway	0.007	39	19
ECM-receptor interaction	1.36E−04	36	19
Long-term depression	0.001	35	19
Dorso-ventral axis formation	0.048	16	19
Oocyte meiosis	0.002	60	18
Inflammatory mediator regulation of TRP channels	0.034	47	18
mRNA surveillance pathway	0.019	46	18
Gap junction	0.001	44	18
Amphetamine addiction	0.010	33	18
Colorectal cancer	0.034	32	18
Gastric acid secretion	0.003	41	17
Melanogenesis	0.013	50	16
Hedgehog signaling pathway	0.010	29	16
Type II diabetes mellitus	0.021	26	16
Circadian rhythm	0.034	20	16
Glycosaminoglycan biosynthesis—heparan sulfate/heparin	0.014	12	12
Fatty acid biosynthesis	3.57E-11	7	11

## Discussion

The results of our current study indicate that several serum miRNAs were regulated in cSCC patients, although only two miRNAs were regulated in AK patients. This is expected as cSCC is a more progressed lesion and more miRNAs were, therefore, regulated. Additionally, our study indicates that there is no differential expressed serum miRNAs between patients with less than 3 or more than 10 AK lesions. Interestingly, we observed that hsa-miR-101-3p was up-regulated in both AK and cSCC lesions, suggesting a common marker between these cells. Conversely, hsa-miR-3168 was up-regulated in AK patients, while strongly down-regulated in cSCC lesions. When comparing our regulated serum miRNAs with a review on the subject we found that hsa-142 and hsa-miR-186 were previously shown to be up-regulated in cSCC tissue samples [31]. Interestingly, hsa-miR-142 was the serum miRNA with the highest fold change in our study. Additionally a previous study has shown that hsa-miR-15b, hsa-let-7i, hsa-miR-140, hsa-miR-101, hsa-miR-16, hsa-miR-22, and hsa-miR-107 were regulated in AK and cSCC tissue samples [16] and we also observed these miRNAs as being regulated in the serum of our patients. Therefore, providing further evidence that a signature of serum miRNAs can be observed in AK and cSCC patients.

miR-142 was 300 times up-regulated in cSCC serum samples. A previous study has shown that miR-142 is up-regulated in cSCC tissue [32] and we previously found that miR-142-3p was up-regulated in tissue samples of head and neck cancer patients [13]. Others observed miR-142–3 as regulated in serum also [33], including as a serum marker of lung cancer recurrence [34]. miR-142 induced cancer stem cells like properties by targeting PTEN [32]. Loss of PTEN function is associated to cancer development [35]. In mice, conditional knockout of PTEN in skin leads to the development of neoplastic lesions [36]. In agreement with these evidences, we observed that signaling pathways regulating pluripotency of stem cells was the top regulated pathway by cSCC miRNA and also regulated by AK miRNAs, further confirming the role of miR-142 in this process. Cancer stem cells (CSCs) have the ability to differentiate in heterogeneous tumor cells, contributing for tumor initiation, growth, and recurrence [37]. Others have shown that the beta-catenin signaling pathway is essential to maintain stem cell-like phenotype in skin cancer [38]. Down-regulation of β-catenin by the proteoglycan decorin, blocks cell scatter, evasion and migration in cancer [39]. Thus, in agreement with our finding that the pathways proteoglycans in cancer was the second most regulated pathway in cSCC lesions.

We observed that miR-15b was up-regulated in serum of cSCC compared to AK patients. Previously miR-15b was observed as up-regulated in melanoma patients [40], suggesting a role for this miRNA in skin cancer. In melanoma tissue samples miR-15b was also observed to be up-regulated [41]. We also observed up-regulation of miR-101-3p in the serum of AK and cSCC patients. Previous studies with cSCC showed that miR-101 was down-regulated in cancer tissue [16]. The same is also observed in breast cancer, in which miR-101 inhibits the growth and proliferation of cancer cells [42]. miR-101-3p targets genes such as VEGF, mTOR, and involved in the focal adhesion, Wnt, and chemokine signaling pathways [43]. In serum, others have also found miR-101 to be down-regulated in cancer patients [44, 45]. More functional studies are needed to better understand the role of miR-101-3p in AK and cSCC.

It surprised us the pattern of regulation for miR-3168. It was up-regulated in early stage AK lesions followed by a strong down-regulation in cSCC samples. Previous work in lung cancer has shown that miR-3168 is target of RNA editing, a common phenomenon that can contribute to development of diseases [46]. The authors observed a decrease in gene editing in early stage tumors followed by increased editing in late stage tumors [46]. Further functional studies are needed in skin cancer samples to confirm how this phenomenon can contribute to the changes in expression levels observed in serum.

We observed that the pathway ‘drug metabolism’—cytochrome P450 was the most enriched pathway in AK patients. The cytochrome P450 is a superfamily of enzymes known as detoxicant enzymes, catalyze the metabolism of several molecules, from lipids and steroidal hormones to xenobiotics [47]. As these enzymes have a role in the metabolism of environmental pollutants, they are involved in the pathogenesis of skin cancer. Ultraviolet-B induction of CYP enzymes in the skin could increase activation of environmental pollutants increasing susceptibility to skin cancers or allergic and irritant contact dermatitis [48]. CYP 450 enzymes are also involved in breast and prostate cancer due its ability to also metabolize steroid hormones [49]. Therefore, suggesting that miRNAs dysregulated in AK patients can contribute to CYP 450 mediated susceptibility to UV damage.

Importantly, based on our published data of circulating miRNAs in head and neck cancer, which allowed us to use differential expression of circulating miRNAs for successful separation of healthy from cancer patients, finding specific miRNAs correlating with different tumor stages, progression, and more importantly patients survival [13, 14, 18, 50], we strongly believe that presented in this study novel findings of differentially expressed circulating miRNAs in patients diagnosed with AK will allow as to use blood circulating miRNAs as non-invasive and easily accessible source of potential biomarkers of AK. More importantly, better understanding of the role of these miRNAs in AK will allow the use of blood circulating miRNAs for early screening for patients with high risk for AK and predicting potential progression towards cSCC in either healthy or already diagnosed patients while using less than 1 ml of blood.

However, we are aware that there are several limitations of the study. The main one is the small study sample and we believe that the presented herein results present strong base for the introduction for further analysis using larger groups and development of more mechanistic studies evaluating the role of circulating miRNAs in AK and cSCC. Additionally, our work is based on analysis of Fitzpatrick II skin types and the skin limited to head location only, and as limiting the selection to one skin type and location represent more homogenous cohort, the inclusion of other skin types and localization would be valuable.

## Conclusion

In sum, we detected a set of 2 miRNAs regulated in AK patients and 12 in cSCC patients. Some of these miRNAs were previously observed as important in AK, cSCC, and other types of skin cancer. Therefore, these miRNAs can be a tool for non-invasive diagnosis of AK and cSCC.
